# Integrative analysis of cancer multimodality data identifying COPS5 as a novel biomarker of diffuse large B-cell lymphoma

**DOI:** 10.3389/fgene.2024.1407765

**Published:** 2024-06-21

**Authors:** Yutong Dai, Jingmei Li, Keita Yamamoto, Susumu Goyama, Martin Loza, Sung-Joon Park, Kenta Nakai

**Affiliations:** ^1^ Department of Computational Biology and Medical Science, The University of Tokyo, Kashiwa, Japan; ^2^ The Institute of Medical Science, The University of Tokyo, Tokyo, Japan

**Keywords:** biomarker discovery, diffuse large B-cell lymphoma, joint non-negative matrix factorization, multi-omics, pathway analysis

## Abstract

Preventing, diagnosing, and treating diseases requires accurate clinical biomarkers, which remains challenging. Recently, advanced computational approaches have accelerated the discovery of promising biomarkers from high-dimensional multimodal data. Although machine-learning methods have greatly contributed to the research fields, handling data sparseness, which is not unusual in research settings, is still an issue as it leads to limited interpretability and performance in the presence of missing information. Here, we propose a novel pipeline integrating joint non-negative matrix factorization (JNMF), identifying key features within sparse high-dimensional heterogeneous data, and a biological pathway analysis, interpreting the functionality of features by detecting activated signaling pathways. By applying our pipeline to large-scale public cancer datasets, we identified sets of genomic features relevant to specific cancer types as common pattern modules (CPMs) of JNMF. We further detected *COPS5* as a potential upstream regulator of pathways associated with diffuse large B-cell lymphoma (DLBCL). *COPS5* exhibited co-overexpression with *MYC*, *TP53*, and *BCL2*, known DLBCL marker genes, and its high expression was correlated with a lower survival probability of DLBCL patients. Using the CRISPR-Cas9 system, we confirmed the tumor growth effect of *COPS5*, which suggests it as a novel prognostic biomarker for DLBCL. Our results highlight that integrating multiple high-dimensional data and effectively decomposing them to interpretable dimensions unravels hidden biological importance, which enhances the discovery of clinical biomarkers.

## 1 Introduction

The era of precision medicine has witnessed a prosperous shift from one-size-fits-all medicine to personalized medicine (Collins and Varmus, 2015). In general, precision medicine aiming for personalized prevention, diagnosis, and treatment requires high-quality biomarkers, which remains challenging (Tsimberidou et al., 2020). Recently, accessible large-scale multimodal data has accelerated the discovery of clinical biomarkers where diverse computational approaches, particularly those integrating multi-omics data, have gained widespread adoption (Bersanelli et al., 2016). On the other hand, the intrinsic nature of biomedical datasets that includes sparse and unlabeled information hinders the practical application of computational methods and limits interpretability and performance (Cho et al., 2023).

Several unsupervised clustering methods have been developed to address these issues and discover potential biological patterns (Reel et al., 2021). For example, the sparse multiple canonical correlation analysis successfully recognized relationships between copy number variations in genomic regions on different chromosomes. Yet, it is infeasible to fully consider the correlation of information across different omics data (Witten and Tibshirani, 2010). Similar Network Fusion (Chiu et al., 2018) identified novel subtypes of triple-negative breast cancer patients but limited applicability to diverse multi-omics scenarios due to its inability to accept multiple data types, such as continuum and binary types. In contrast, joint non-negative matrix factorization (JNMF), an unsupervised algorithm, complements the bottlenecks in those methods and affords to extract underlying features from sparse high-dimensional heterogeneous data (Zhang et al., 2012; Yang and Michailidis, 2016).

In this study, we aim to develop a method to discover interpretable biomarkers from intricate multimodal data. To this end, we designed a novel pipeline that integrates JNMF and a biological pathway analysis; the functionality of JNMF-detected genetic features is implicated through detecting signaling pathways specifically activated by the features. We demonstrate the ability to find reliable biomarkers from the large-scale cancer datasets of the Cancer Cell Line Encyclopedia (CCLE) and The Cancer Genome Atlas (TCGA). In particular, we identified *COPS5* as a novel biomarker for diffuse large B-cell lymphoma (DLBCL) and experimentally validated by the CRISPR-Cas9 knockout, which supports the feasibility of our approach.

## 2 Materials and methods

### 2.1 Data preparation and processing

The raw dataset of the CCLE project (DepMap release 23Q4) was downloaded *via* the DepMap portal (https://depmap.org/portal/). The dataset consisted of six matrices including gene expression, CNV (copy number variation) amplification, CNV loss, DNA mutation of somatic point mutations and indels, pharmacologic sensitivity, and metadata of cell lines. For correlation analysis and survival analysis, the expression profiles of DLBCL patients were downloaded from TCGA (https://www.cancer.gov/tcga) and NCBI Gene Expression Omnibus (GEO) GSE69049.

We prepared the CCLE datasets only for 504 cell lines presented in the pharmacologic sensitivity matrix and built the input matrices of JNMF as follows. The TPMs (transcripts per million) on a log-2 scale quantifying gene expressions were converted ranging from 0 to 1 by a min-max normalization. The DNA mutation profile was converted into a binary matrix where 1 for mutated and 0 for normal. Two binary matrices for CNV gain and loss were constructed by the GISTIC scores in the CNV data; +2 for amplification and −2 for deletion. The pharmacologic sensitivity was also converted into values ranging from 0 (insensitive) to 1 (sensitive) as follows:

$$\max\left(X\right)\;–\;x\;/\;\max\left(X\right)\;–\;\min\left(X\right),$$

where *x* is an IC50 (half maximal inhibitory concentration) value and max(X) and min(X) are the maximum and minimum values in the pharmacologic sensitivity profile. A cancer-type matrix in binary format was prepared from the metadata of cell lines.

### 2.2 Plasmids and viral infection

To generate single-guide RNA (sgRNA) expression vectors targeting *COPS5* (*COPS5*-sgRNA-1 and *COPS5*-sgRNA-2) or a non-targeting (NT) control, annealed oligonucleotides were cloned into the pLKO5.sgRNA.EFS.tRFP657 vector (Addgene plasmid # 57824; http://n2t.net/addgene:57824), which was a gift from Benjamin Ebert. The Cas9 expression in the cell lines Raji (Burkitt lymphoma cell line) and SLVL (Splenic marginal zone lymphoma cell line) was induced by FUCas9Cherry plasmid, which was a gift from Marco Herold (Addgene plasmid # 70182, http://n2t.net/addgene:70182). Lentiviruses were produced by transient transfection of 293T cells with viral plasmids, along with gag-, pol-, and env-expressing plasmids (pMD2.G and psPAX2) using the calcium-phosphate method (Goyama et al., 2016). pMD2.G (Addgene plasmid #12259; http://n2t.net/addgene:12259) and psPAX2 (Addgene plasmid #12260; http://n2t.net/addgene:12260) were gifts from Didier Trono. The sequences for the sgRNAs are as follows: NT: 5′-cgc​ttc​cgc​ggc​ccg​ttc​aa-3′, *COPS5*-sgRNA-1: 5′-gtg​atg​cat​gcc​aga​tcg​gg-3′, *COPS5*-sgRNA-2: 5′-caa​caa​gaa​caa​tat​ccg​ca-3′.

### 2.3 Cell culture and CRISPR/Cas9-mediated gene knockout

The lymphoma cell lines Raji and SLVL were cultured in RPMI1640 medium supplemented with 10% fetal bovine serum (FBS) and 1% penicillin. 293T cells (CRL-11268, ATCC, Manassas, VA, United States) were cultured in Dulbecco’s modified Eagle’s medium supplemented with 10% FBS and 1% penicillin. These cells were first transduced with the FUCas9Cherry, followed by sorting of mCherry^+^ cells using BD FACSAriaIII (BD Biosciences, San Jose, CA, United States). The Cas9-expressing (mCherry^+^) Raji and SLVL cells were then transduced with the sgRNAs co-expressing tRFP657. The frequency of tRFP657^+^ cells in the cultures was evaluated on Day3, Day6, Day10, Day17 and Day24.

Cells were sorted by FACS Aria (BD Biosciences, San Jose, CA, United States), and the expression of mCherry and tRFP657 was analyzed with FACS CytoFLEX (Beckman Coulter, Brea, California, United States). The cytometry data were analyzed by BD FlowJo software (TREESTAR, Inc., San Carlos, CA. ver.10.8.1).

### 2.4 Joint non-negative matrix factorization (JNMF)

JNMF, an extension of traditional NMF algorithm, is designed to facilitate the simultaneous decomposition of *N* datasets (Zhang et al., 2012). The objective function of JNMF is given by:

$$\min\sum_{i=1}^{N}{\left\|X_{i}-{WH}_{i}\right\|}_{F}^{2},$$

where *X*_*i*_ is an input matrix with size *m*×*n*_*i*_, and *F* represents the Frobenius norm. The *W* and *H*_*i*_ represent *m*×*k* and *k*×*n*_*i*_ factorized matrices, respectively. Here, *k* is the number of clusters to be extracted, namely, common pattern modules (CPMs). To find the optimal *W* and *Hi* minimizing the objective function, JNMF updates them based on the traditional multiplication update formulas (Zhang et al., 2012) as follows:

$$W_{ia}=W_{ia}\frac{{\left(\sum_{J=1}^{N}X_{J}H_{J}^{T}\right)}_{ia}}{{\left(W\sum_{K=1}^{N}\left(H_{K}H_{K}^{T}\right)\right)}_{ia}}$$

$${\left(H_{I}\right)}_{a\mu }={\left(H_{I}\right)}_{a\mu }\frac{{\left(W^{T}X_{I}\right)}_{a\mu }}{{\left(W^{T}{WH}_{I}\right)}_{a\mu }},I=1,\cdots ,N.$$

To handle missing values in the input matrices, we employed a weighted NMF approach (Zhang et al., 2012): a mask matrix *M* representing 1 for non-missing and 0 for missing cases is introduced into the JNMF framework. *X* is accessed by the Hadamard product with *M* effectively filtering the influence of missing values (Fujita et al., 2018). The objective function and multiplicative update rules of the weighted JNMF are given by:

$$\min\sum_{i=1}^{N}{\left\|{M_{i}\circ X}_{i}-{WH}_{i}\right\|}_{F}^{2}$$

$$W_{ia}=W_{ia}\frac{{\left(\sum_{J=1}^{N}\left({M_{i}\circ X}_{J}\right)H_{J}^{T}\right)}_{ia}}{{\left(\sum_{K=1}^{N}\left(M_{i}\circ \left({WH}_{K}\right)H_{K}^{T}\right)\right)}_{ia}}$$

$${\left(H_{I}\right)}_{a\mu }={\left(H_{I}\right)}_{a\mu }\frac{{\left(W^{T}\left({M_{i}\circ X}_{I}\right)\right)}_{a\mu }}{{\left(W^{T}\left({M_{i}\circ WH}_{I}\right)\right)}_{a\mu }},I=1,\cdots ,N,$$

where $A\circ B=\left[a_{ij}b_{ij}\right]$ is Hadamard product.

### 2.5 Hyperparameter optimization

Given a factorization rank *k*, JNMF starts with random *W* and *Hs* and updates the random matrices toward minimizing the objective function for *n* iterations step-by-step. To tune the hyperparameters, whether the procedure with *k* and *n* stably convergent in repeating *t* times is monitored. A consensus matrix and its cophenetic correlation coefficient (CCC) (Brunet et al., 2004) evaluate the performance of JNMF under the setting of *k* and *n*.

### 2.6 Selection of features for each CPM in each H matrix

In analyzing the six input matrices of CCLE datasets, our JNMF produces seven matrices: *W*, *H*_*1*_, *H*_*2*_, *H*_*3*_, *H*_*4*_, *H*_*5*_, and *H*_*6*_. For normalizing the matrices, rather than using the maximum values, z-score normalization was applied to each row and column of *W* and *H*s as follows:

$$z_{ij}=\frac{x_{ij}-\mu_{i}}{\sigma_{i}},$$

where $\mu_{i}$ and $\sigma_{i}$ stand the average and the standard deviation for cell line/drug/mutation/CNV/genes/cancer type feature *j*, respectively. The feature *j* is assigned to CPMs if and only if $z_{ij}$ is > +1.96.

### 2.7 Pathway analysis

To identify the activated pathways within each CPM obtained from JNMF, we employed IPA (Ingenuity Pathway Analysis) (Krämer et al., 2014). IPA is a widely used tool for exploring signaling pathways and biological networks. Specifically, IPA’s upstream analysis and protein-protein interaction analyses were performed to determine general regulators in CPM-activating pathways.

### 2.8 Correlation analysis

For examining the correlation between candidate biomarkers and known hub genes of DLBCL, we utilized GEPIA2 (Gene Expression Profiling Interactive Analysis 2) (Tang et al., 2019). GEPIA2 is a comprehensive web-based tool that integrates data from the TCGA and GTEx databases. Specifically, we used the “correlation analysis” module within GEPIA2, utilizing the TCGA-DLBCL project dataset, to assess the correlation between the candidate biomarkers and the known hub genes of DLBCL.

### 2.9 Survival analysis

The Kaplan-Meier method was used to investigate whether the candidate biomarkers in GSE69049 datasets affected the overall survival (OS) of DLBCL patients treated with chemotherapy. The “survminer” R package was used to explore survival analysis.

## 3 Results

### 3.1 Overall workflow of the proposed method

**FIGURE 1:**
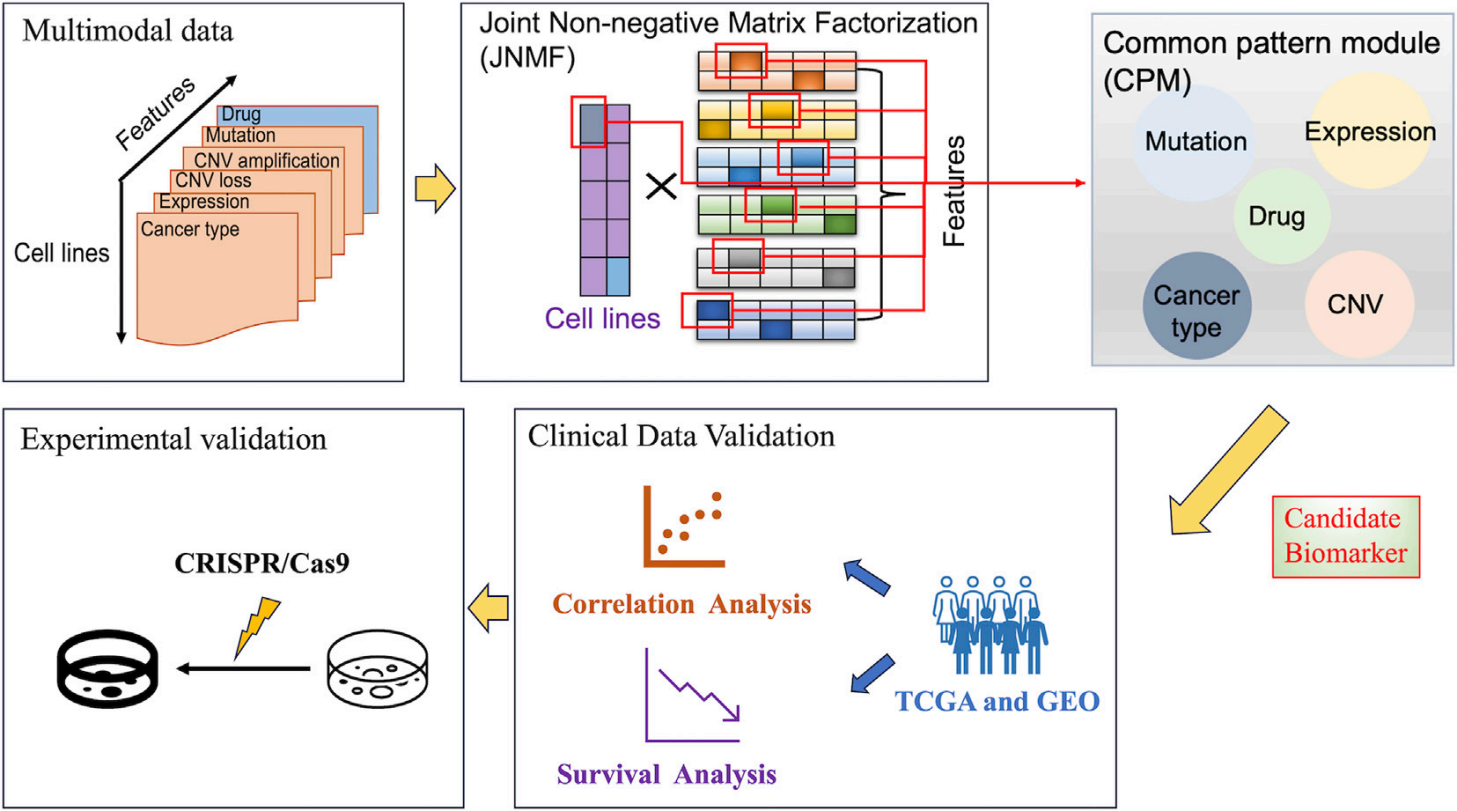
Overall workflow of our analysis. The figure outlines a systematic approach for identifying candidate biomarkers in cancer research, starting with the collection of multimodal data. These data were analyzed using joint non-negative matrix factorization to identify common pattern modules and combined with pathway analysis to highlight potential biomarkers at the intersection of different data types. Then, these biomarkers were clinically validated by correlation and survival analyses using TCGA and GEO data, and lastly, experimentally validated using CRISPR/Cas9 system to determine their promising applications in cancer cells.

We designed a method to find significant sets of multimodal factors (i.e., modules) that have the potency to characterize disease phenotypes. Since multimodality comprises multiple high-dimensional heterogeneous data, we adopted JNMF, a well-proven algorithm for clustering the factors as modules, by modifying it to handle data sparseness efficiently, referred to as weighted JNMF. To interpret the functional importance of the JNMF-detected modules, namely, the common pattern module (CPM), we utilized a pathway analysis by detecting upstream regulators in signaling pathways activated by the modules (Figure 1).

To perform the biomarker discovery using the proposed method, we complied six feature matrices for 504 cancer cell lines by processing the large-scale CCLE datasets, each of which has binary or continuous values: gene expression, CNV amplification, CNV loss, DNA mutation, pharmacologic sensitivity, and cancer type (Supplementary Table S1). Using these matrices as inputs, the weighted JNMF masks sparse elements and generates a factor matrix *W* given by latent coefficients for the cancer types in the reduced *k*-dimensional space (i.e., rank). Simultaneously, the six input matrices are reduced to the *k* dimension generating the matrices *H*_*i*_ (*i* = 1, … ,6), where each product with *W* approximates the original input matrix. Given optimal *W* and *H*s, significant feature sets (>+1.96 in z-score) are captured as CPMs by investigating z-score distributions in the factorized matrices.

Subsequently, the gene expression profiles in the JNMF-detected CPMs are analyzed by IPA to identify activated pathways. Meanwhile, the disease subtypes corresponding to the CPM are identified by combining characteristic drugs, DNA mutations, and structural variants in the CPM. Moreover, through IPA upstream analysis and IPA causal network analysis, the upstream regulators of CPMs which are considered candidate biomarkers are investigated. Next, the correlation and survival analyses of candidate biomarkers using TCGA and GEO data examine the clinical significance of the candidate biomarkers. Ultimately, the candidate biomarkers are experimentally validated by the CRISPR-Cas9 system.

### 3.2 Assessing the robustness of JNMF

**FIGURE 2:**
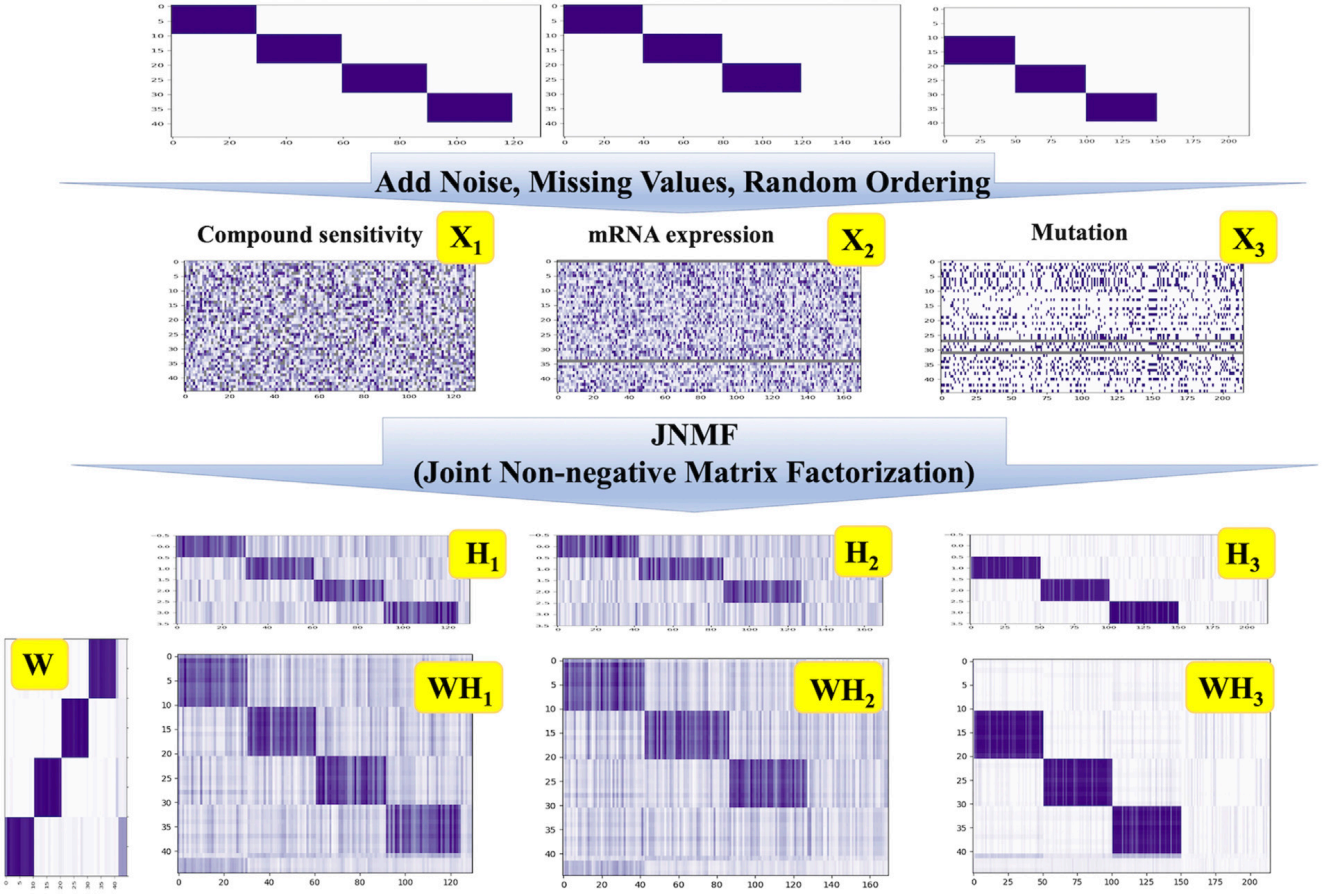
JNMF clustering results of simulated data. JNMF was utilized to identify CPMs embedded in simulated pharmacologic sensitivity, mutation, and expression matrix. The continuous simulated pharmacologic sensitivity matrix X_1_ comprises four modules alongside missing values. The continuous simulated expression matrix X_2_ comprises three modules alongside missing rows. The binary simulated mutation matrix X_3_ comprises three modules alongside missing rows. The Gaussian noise is introduced into X_1_ and X_2_ matrix. The value in X_3_ is partially reverse as the noise. The grey parts represent the missing value.

To interrogate the ability of our JNMF, we prepared three artificial datasets as used in a previous study (Fujita et al., 2018). We first constructed three matrices with random values imprecating noise: a binary matrix for mutation and continuous matrices for pharmacologic sensitivity and gene expression. Then, we inserted missing values into randomly selected 10% of the entries of each matrix: the missing rates in CCLE datasets were 2.6%–10.5%. Next, we embedded three or four predefined CPMs into the matrices and randomly shuffled the entries in each matrix. Thereby, three artificial input data that are noisy and sparse but include modules were generated (Supplementary Table S2, Figure 2).

Since the embedded CPMs that our JNMF has to detect were three or four, we set the rank *k* = 4. As shown in Figure 2, our JNMF successfully identified the CPMs by decomposing the input matrices into *W*, *H*_*1*_, *H*_*2*_, and *H*_*3*_. In addition, the products *WH*_*1*_, *WH*_*2*_, and *WH*_*3*_ accurately restored the input matrices. This result demonstrates that our JNMF can uncover hidden relationships within high-dimensional multimodal datasets by reducing the influence of noise and missing values.

### 3.3 Identifying CPMs corresponding to cancer types

**FIGURE 3:**
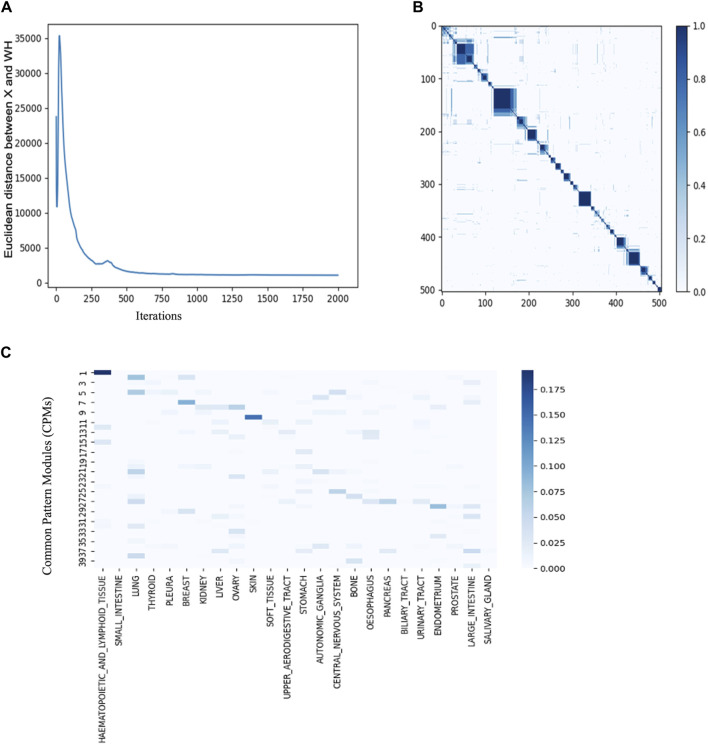
JNMF identifies biological features in multimodal data **(A)** Convergence curve showing the trajectory of JNMF objective function convergence under 2000 iterations **(B)** Consensus matrix of W showing the reproducibility of JNMF result in 10 repeated trials. The blue rectangles represent clusters of the cell lines highly reproduced. **(C)** The distribution of cancer types in CPMs. The blue rectangles represent the specificity of each cancer in the CPMs. Darker blue corresponds to stronger specificity. CPM #1 shows high specificity for hematopoietic and lymphoid malignancies.

To optimize the hyperparameters of JNMF for the six input matrices prepared from CCLE gene expression, CNV amplification, CNV loss, DNA mutation, pharmacologic sensitivity, and cancer cell line type, we investigated the convergence of JNMF during 2000 iterations updating W and Hs (Figure 3A). We noticed that the outputs of JNMF are stable (CCC = 0.72) when *k* = 40 and manifest substantial consistency across 10 repeats (Figure 3B). Using these hyperparameters, we finally retrieved 40 CPMs corresponding to specific cancer types, such as CPM #1 for hematopoietic and lymphoid malignancies, CPM #7 for breast cancer, CPM #10 for malignant melanoma, and CPM #28 for endometrial cancer (Table1; Figure 3C).

**TABLE 1:** Summary of key features in CPMs.

CPM	Drug in CPM	Genetic features in CPM	Cell lines in CPM
CPM #1	AEW541	Mutation (BCL2, BCL6, IGF1R, MTOR, MYC, MYD88, PI3KR1, PTEN, SPEN, STAT3, TP53)	Lymphoma
CPM #7	Lapatinib	BRACA2 mutation, HER2 amplification, HER2 overexpression	Breast Cancer
CPM #10	PLX4720	BRAF mutation, MITF amplification	Skin Cancer
CPM #28		TP53 mutation, PTEN mutation, Overexpression (MLH1, MSH2, MSH6, and PMS2)	Endometrium cancer

The CPM #1 contained *TP53* mutation and the malignant translocations of *MYC*, *BCL2*, and *BCL6*, which are relevant to DLBCL (Chapuy et al., 2018). The CPM #7 included the pharmaceutical agent Lapatinib, *HER2* overexpression, and *BRCA2* amplification, which is supported by the clinical application of Lapatinib for treating *HER2*-positive metastatic breast cancer (Xu et al., 2021). The CPM #10 covering almost the entire skin cancer cell lines included the pharmaceutical agent PLX4720 (vemurafenib) and *BRAF* mutation; the efficacy of vemurafenib has been tested in several clinical trials for treating unresectable or metastatic melanoma with *BRAF* V600E mutation (Chapman et al., 2011). The CPM #28 confined several known diagnostic and prognostic biomarkers of endometrial cancer, such as the mismatch repair mutation, and the overexpression of *MLH1*, *MSH2*, *MSH6*, and *PMS2*. In addition, this module included the overexpression and mutation of *PTEN* and *TP53*, which significantly contribute to the diagnosis of endometrial cancer (Crosbie et al., 2022).

Collectively, we confirmed that the CPMs are in high concordance with known relationships among variants, medications, and cancers, which suggests the potency of our approach to the discovery of novel biomarkers from multimodal data.

### 3.4 Analyzing biological pathways activated by DLBCL-related CPM

To interpret the functionality of JNMF-detected CPMs, we focused on the CPM #1 that includes DLBCL biomarkers (Table 1). Notably, this module also contained several gene mutations, each of which is known to be involved in pathogenic pathways: *MYD88* and *CARD11* functioning in the NF-κB pathway, *SPEN* involved in the NOTCH signaling, and *STAT3* which is a pivotal member of the JAK/STAT signaling pathway (Chapman et al., 2011).

Next, we performed a pathway analysis of IPA with the gene expression profile of CPM #1. Consistent with the pathways in which the mutated genes are involved, we found the activation of several signaling pathways of DLBCL (*p* < 0.05). For example, the activation of the NF-κB pathway causing DLBCL (Odqvist et al., 2014), the deregulation of the JAK-STAT pathway and PI3K-mediated signaling pathway which is the essential contributor to the pathogenesis and poor prognosis of DLBCL (Chapuy et al., 2018) (Supplementary Figure S1). In addition, the sub-networks centered on *MYC*, *TP53*, and NF-κB indicated the activation of downstream pathways potentially relevant to DLBCL development (Supplementary Figure S2). Lastly, by performing the IPA upstream analysis and IPA causal network analysis, we investigated upstream regulators (*p* < 0.05) likely controlling the gene expression of CPM #1 (Tables 2).

**TABLE 2:** Top five genes in IPA upstream regulators and causal network analyses.

IPA upstream regulators analysis		IPA causal network analysis	
Gene	*p*-value	Gene	*p*-value
COPS5	2.58E-25	COPS5	1.06E-25
E2F4	1.38E-20	TFEB	4.23E-16
UQCC3	7.53E-18	NUPR1	3.80E-14
TFEB	4.23E-16	BCR	4.31E-11
NUPR1	2.57E-14	RPL11	3.09E-10

**FIGURE 4:**
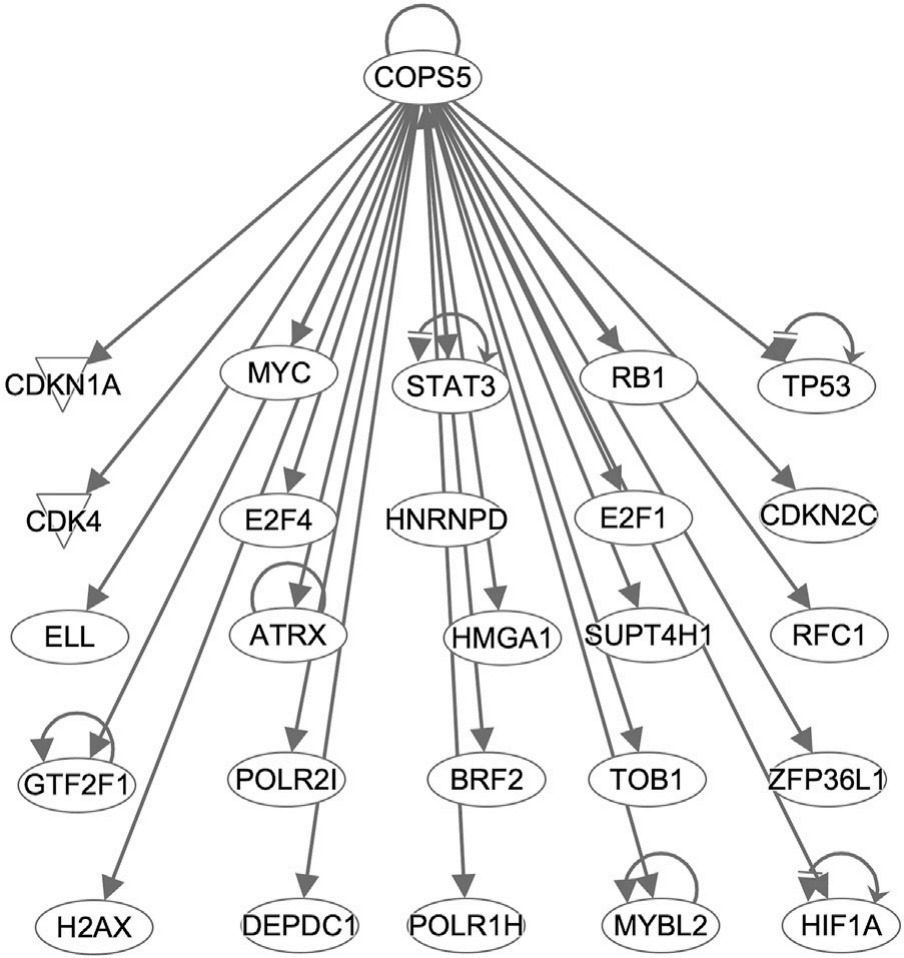
COPS5-targetting genes in the CPM #1. Arrows indicate that *COPS5* directly regulates the expression of connected genes.

Interestingly, *COPS5*, a subunit of the COP9 signalosome complex, was identified from both analyses and showed regulatory interactions with the overexpressed genes in the CPM #1 including known DLBCL markers (Figure 4). This result supports that *COPS5* overexpression affects tumor-negative regulators in diverse cancers (Wang et al., 2016). For example, *COPS5* activates *MYC* by mediating the SKP1-CUL1-F-box protein complex (Lyapina et al., 2001). Also, *COPS5* alters cytoplasmic localizations of *TP53* and induces the degradation of *TP53* (Li et al., 2013). Moreover, *COPS5* is co-expressed with *STAT3* in cancers (Nishimoto et al., 2013), and *MYC* mediates this co-regulation associated with poor prognosis (Ok et al., 2014).

Taken together, despite the considerable heterogeneity among DLBCL subtypes (Schmitz et al., 2018), the CPM #1 collectively retained the distinctive characteristics of DLBCL, which emphasizes the importance of understanding the orchestration of multiple oncogenic factors. Moreover, we identified *COPS5* using the information of this module as a potential upstream regulator of DLBCL, requiring further validations.

### 3.5 Inferring the impact of COPS5 in DLBCL

**FIGURE 5:**
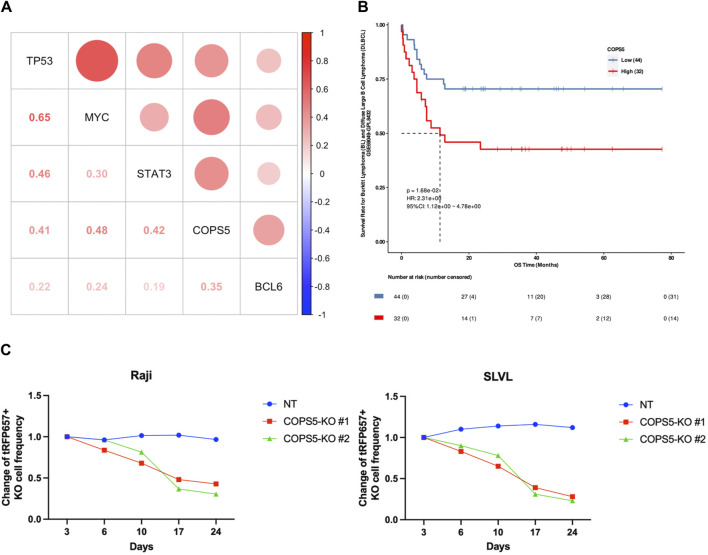
**(A)** The correlation scatter plots between CPM #1 overexpression genes in DLBCL patients. **(B)** Kaplan-Meier curve indicating the effect of high (red) and low (blue) expression of *COPS5* on the overall survival (OS) of DLBCL patients. **(C)** Changes in the frequency of Raji and SLVL cells cultured with *COPS5* depletions compared to controls (NT). The cells were transduced with Cas9 together with non-targeting (NT) or *COPS5*-targeting sgRNAs (*COPS5*-KO#1 and *COPS5*-KO #2) co-expressing tRFP657. The frequency of tRFP657+ cells was normalized to the frequency of tRFP657+ cells at day 3.

Using the gene expression profiles of 47 DLBCL patients available at TCGA, we sought to confirm the importance of *COPS5* in DLBCL patients. Consistent with the results of CCLE data analysis, we observed the positive expression correlation between *COPS5* and the marker genes, as well as between *TP53* and *MYC* (Figure 5A). It is noteworthy to mention that all the positive correlations have been reported by previous *in vitro* studies (Adler et al., 2006; Sitte et al., 2012; Li et al., 2013; Nishimoto et al., 2013; Luo et al., 2022). Interestingly, even *BCL6*, *MYC*, and *TP53* were grouped in the CPM #1, their expression correlations in the patients were relatively less as shown in Figure 5A, E.g., R = 0.22 between *BCL6* and *TP53*, R = 0.24 between *BCL6* and *MYC*. This result might reflect the high heterogeneity of DLBCL. Therefore, *COPS5* may be a key hub gene that efficiently characterizes heterogeneous DLBCL by co-expressing with the marker genes.

Next, we performed the survival analysis to inspect the relationship of *COPS5* with the prognosis of DLBCL patients. As shown in Figure 5B, the Kaplan-Meier curve exhibited that the high expression of *COPS5* is associated with poor prognosis in the overall survival of the patients treated with chemotherapy alone (*p* = 0.0168). This result supports that the over expression of *COPS5* promotes malignancy.

Finally, Since *COPS5* has been shown to be associated with the proliferation of DLBCL in several cell lines derived from subtypes of DLBCL (Pulvino et al., 2015), to further determine the role of *COPS5* in B cell lymphoma, we depleted *COPS5* in the B cell lymphoma cell lines Raji and SLVL using the CRISPR/Cas9 system. Raji and SLVL cells were transduced with Cas9 together with tRFP657-coexpressing non-targeting (NT) or *COPS5-*targeting sgRNAs. As shown in Figure 5C, *COPS5* depletion showed a strong growth-inhibitory effect in Raji and SLVL cells. These results confirm the key role of *COPS5* in the proliferation of malignant B cells (Supplementary Figure S3).

## 4 Discussion

In this study, we proposed a JNMF-based method that integrates sparse multimodal data and reduces their higher dimensionality into interpretable lower dimensions. Our method captures CPMs grouping potentially relevant heterogeneous modalities and utilizes IPA for interpreting the biological importance of CPMs. Hence, contrary to traditional correlation-based predictions, our approach combines machine learning results with biological knowledge for rigorous inference, designed to uncover hidden relationships within intricate multimodality.

We applied the method to speculate key factors responsible for drug sensitivity in various cancers using CCLE datasets which provide plenty of genotype and phenotype annotations. Consequently, we successfully retrieved CPMs comprising CNVs, genomic mutations, and medications, characterizing the cancer types: for example, a metastatic breast cancer-related module showing the known relationship among the drug Lapatinib and gene mutations on *HER2*, and *BRCA2*, and a skin cancer-related module containing the BRAF-inhibitor PLX4720 and *BRAF* mutation. Unexpectedly, the CPM related to lymphoma contained *COPS5* co-overexpressing with DLBCL marker genes, e.g., *MYC*, *TP53*, and *STAT3*, and located upstream of the relevant pathways. The functional importance of *COPS5* was also confirmed in DLBCL patients and knockout experiments, revealing the significant contribution to poor prognosis and cancer cell proliferation. Constrained by the number of patients in the database, this result may need to be further validated in a larger scale of patient data.

It has been reported that the expression of *COPS5* is a prerequisite for the *MYC* activity in breast cancer (Hou et al., 2017). On the other hand, *COPS5* or *MYC* alone is insufficient to activate genes crucial for tumor growth and invasion fully (Adler et al., 2006), indicating their cooperativity is indispensable in cancer development. Regarding the tumor suppressor *TP53* co-overexpressing with *COPS5* in the DLBCL-related CPM, since the CPM contained *MYC* and *TP53* mutations also, our results suggest the importance of understanding the orchestration of multimodal features. Indeed, it has been reported that patients with overexpression of *TP53* in the presence of *TP53* mutations display chemotherapy resistance and poor prognosis (Li et al., 2013). We expect that our CPM, particularly *COPS5*, collectively explains this gain-of-function mutation, which needs further investigation.

We recognize that our current model input matrices do not include delicate genomic features, including various structural variants, mutation zygosity, and gene colocalization analysis. Enhancing our model by incorporating these additional patterns and refining preprocessing steps might improve the JNMF outcomes, allowing us to reveal more intricate biological relationships. Such improvements would expand the depth and breadth of our methodology. Despite these limitations, the features currently included have successfully identified biologically meaningful biomarkers, demonstrating our approach’s robust scalability. This validation underscores the reliability of our model and underscores its potential for adaptation and growth with the integration of new data and advanced techniques.

In conclusion, our integrative analysis handled the sparsity of large-scale multimodal datasets by effectively decomposing them and offers the functional relationships among the high-dimensional features in disease phenotypes. Our findings highlight that integrating complementary data will facilitate clinical biomarker discovery, greatly advancing precision oncology.

## Data Availability

The original contributions presented in the study are included in the article/Supplementary Material, further inquiries can be directed to the corresponding author.
