# Supplementary material for: Integrative analysis of cancer multimodality data identifying COPS5 as a novel biomarker of diffuse large B-cell lymphoma
Source: Front Genet. 2024 Jun 21;15:1407765. doi: 10.3389/fgene.2024.1407765 (PMC11224480; doi:10.3389/fgene.2024.1407765)
Supplement: Supplementary file 1 [file DataSheet1.ZIP › Supplementary_Material_v1.docx]

Supplementary Material

Supplemental Material includes:

# Supplementary figures

**Supplementary Figure 1.** Snapshot of JAK-STAT signaling pathway and PI3K-mediated signaling pathway map.

**Supplementary Figure 2.** MYC, TP53, NF- κB gene subnetworks in CPM1#.

**Supplementary Figure 3.** Examples of flow cytometry images

# Supplementary tables

**Supplementary Table 1.** The detail of 6 preprocessed input matrices.

**Supplementary Table 2.** the detail of simulated matrices.

# Supplementary figures


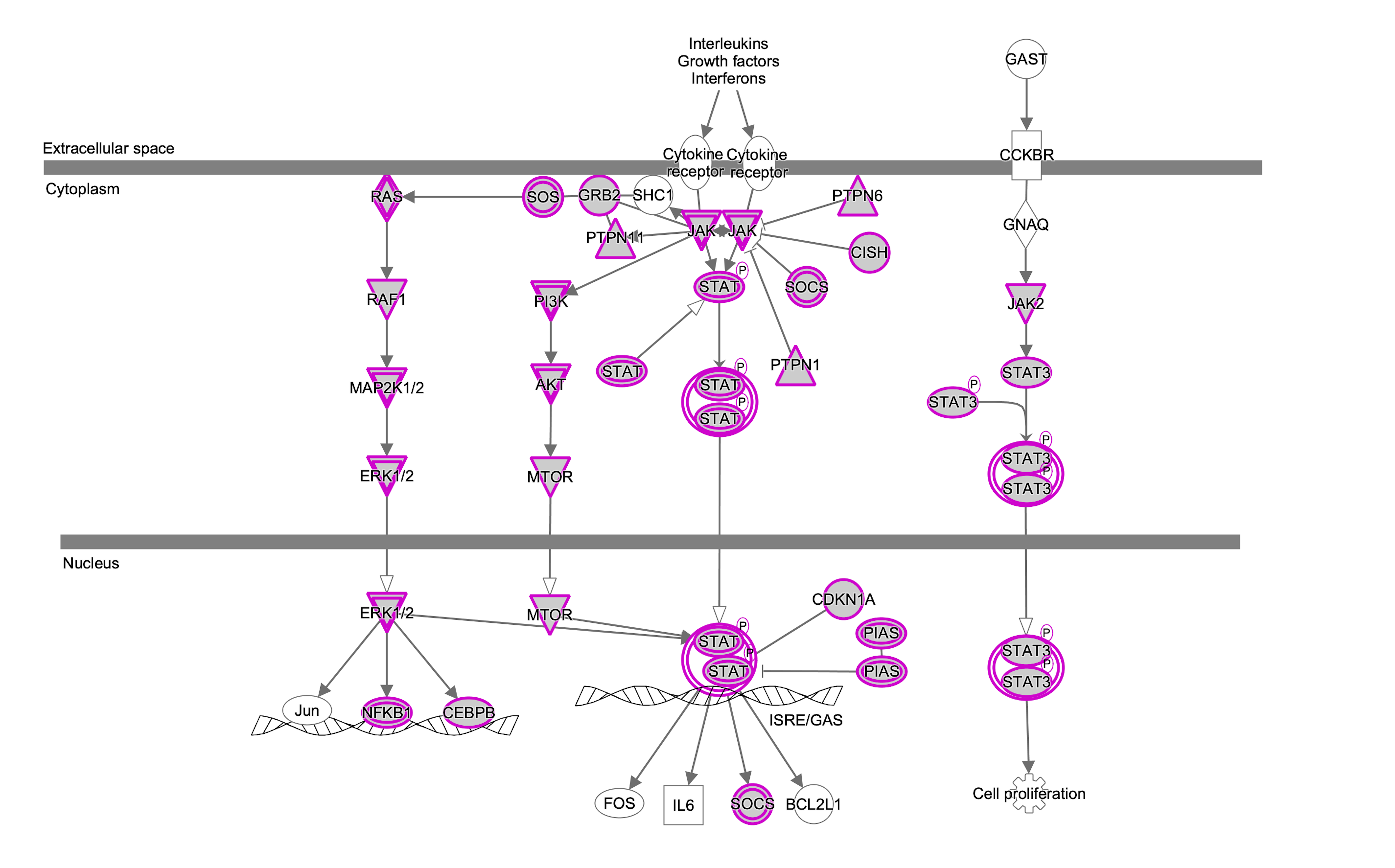


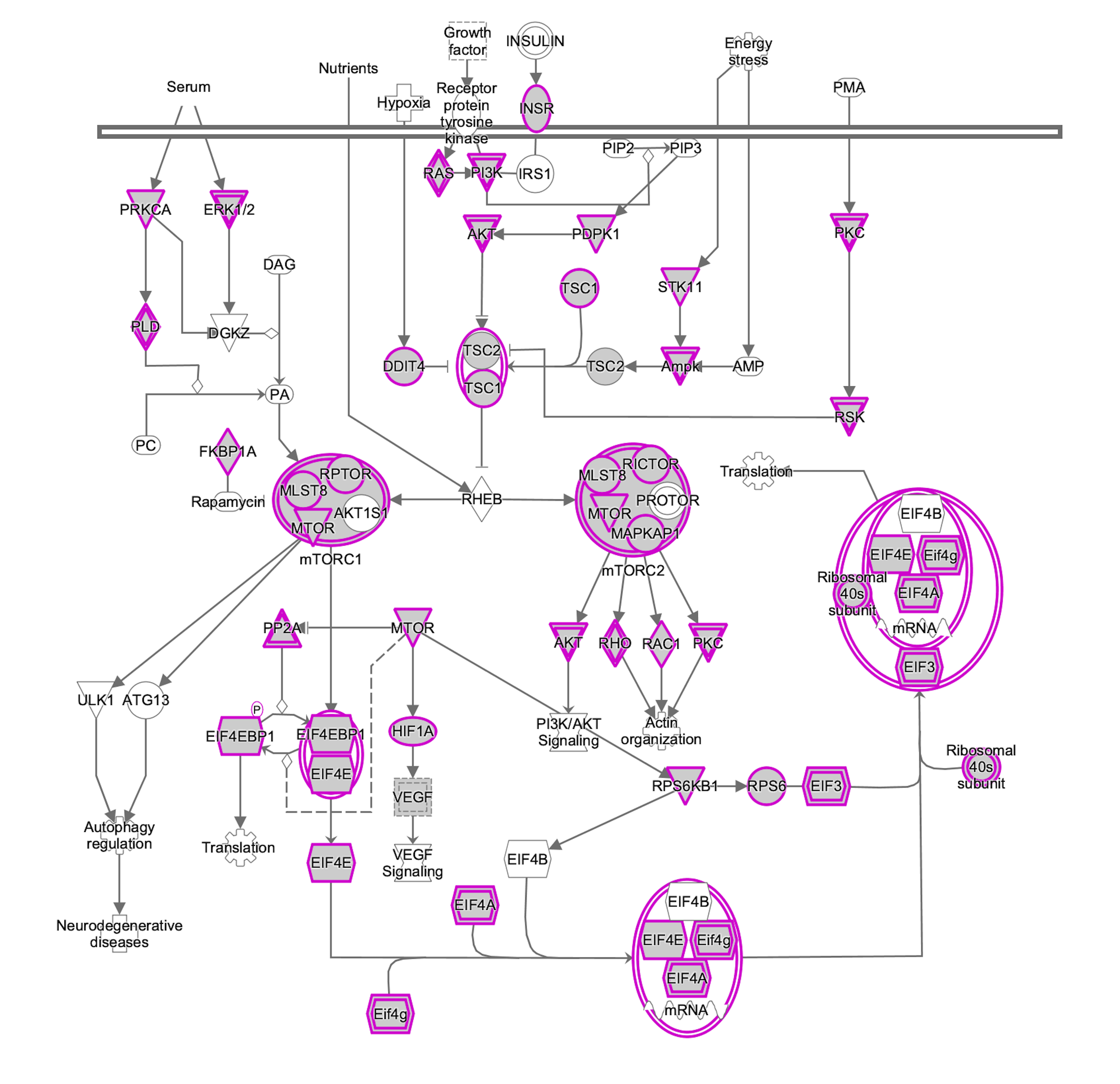


Supplementary Figure.1 Snapshot of JAK-STAT signaling pathway and PI3K-mediated signaling pathway map. Genes selected into CPM #1 are highlighted in gray parts.


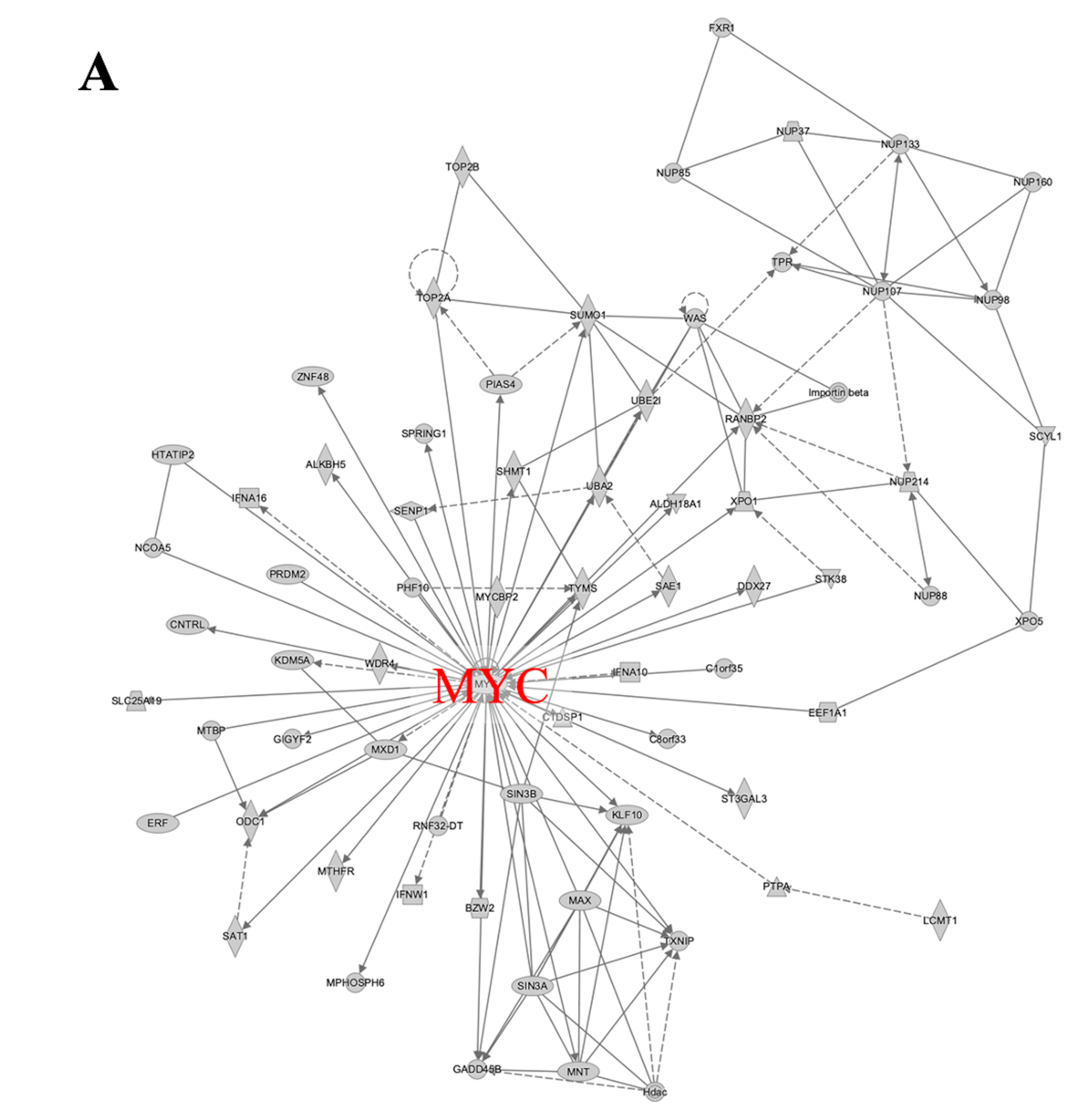


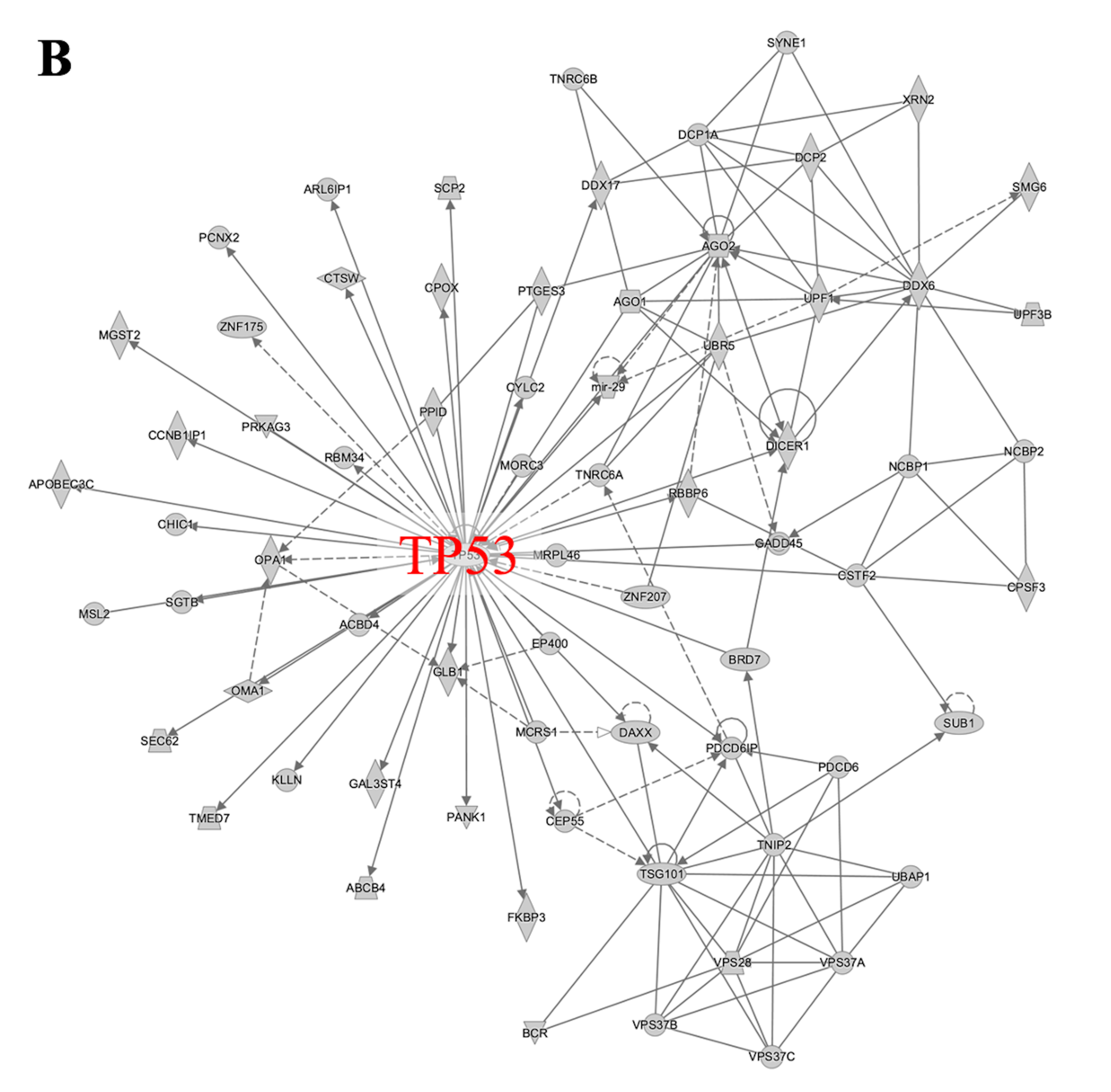


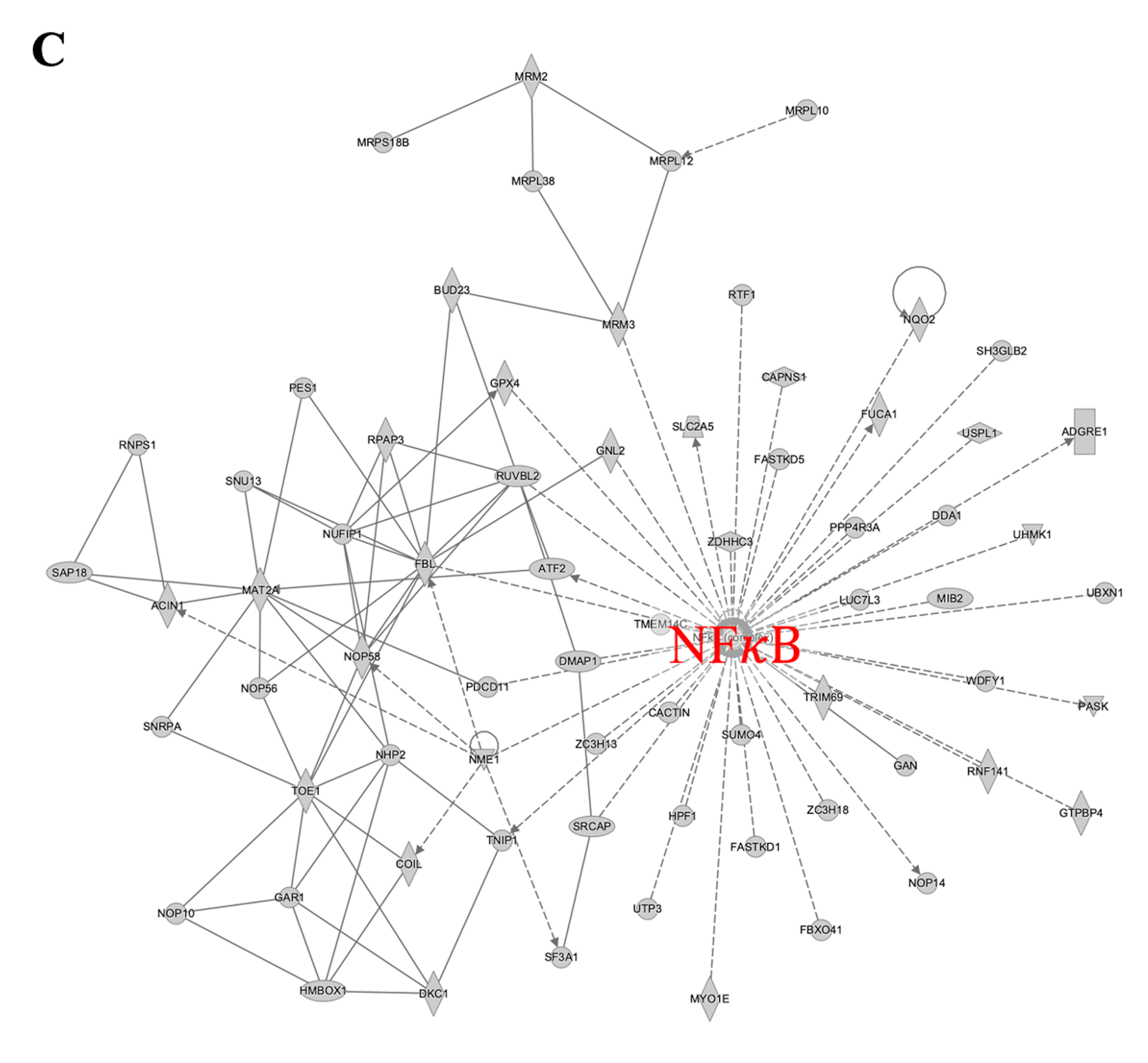


Supplementary Figure.2 MYC, TP53, NF- κB gene subnetworks in CPM #1. (A) MYC; (B) TP53; (C) NF- κB


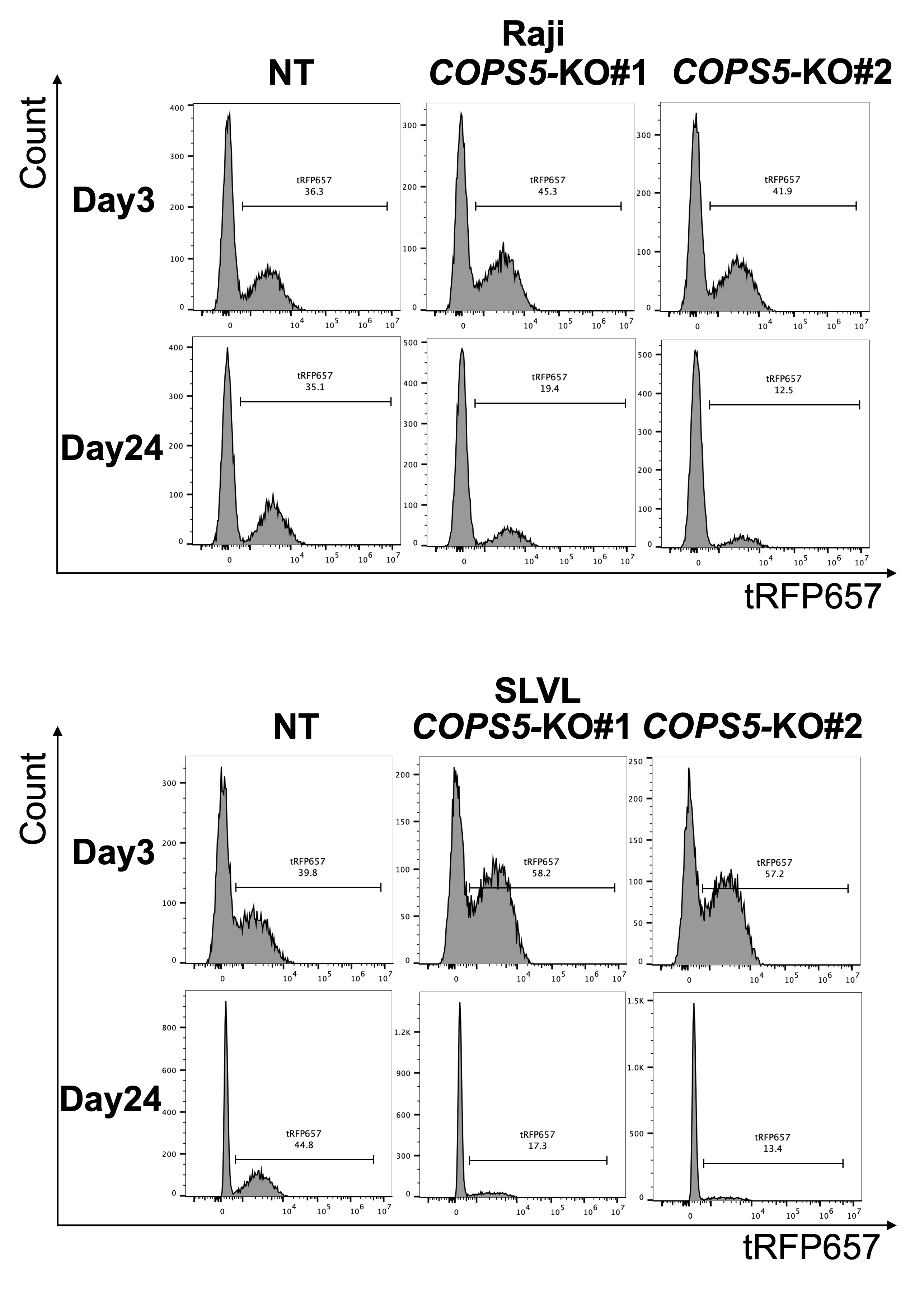


Supplementary Figure.3 Representative images of Raji and SLVL cells transduced with Cas9 together with either non-targeting (NT) sgRNAs or COPS5-targeting sgRNAs (COPS5-KO#1 and COPS5-KO#2), co-expressing tRFP657 at day 3 and day 24. See also Figure.5C

# Supplementary tables

| Input Matrix | Number of Features | Feature Type | Number of Cell Lines | Missing Rate |
| --- | --- | --- | --- | --- |
| Pharmacologic sensitivity | 24 | Continuous | 504 | 3.52% |
| Mutation | 4090 | Binary | 504 | 10.51% |
| CNV Amplification | 16384 | Binary | 504 | 3.17% |
| CNV Loss | 16384 | Binary | 504 | 3.17% |
| Expression | 16384 | Continuous | 504 | 2.58% |
| Cancer Type | 24 | Binary | 504 | 0.00% |

Supplementary Table 1 The detail of 6 preprocessed input matrices; In public databases, not all cell lines have corresponding drug susceptibility, transcriptomic and genomic data, so the missing rate here represents the proportion of each dataset that does not have corresponding data for a cell line.

| Input Matrix | Feature | Feature Type | Cell Lines | Missing Rate |
| --- | --- | --- | --- | --- |
| Drug | 130 | Continuous | 45 | 10% |
| Mutation | 215 | Binary | 45 | 10% |
| Expression | 170 | Continuous | 45 | 10% |

Supplementary Table 2 the detail of simulated matrices
